# The Epimed Monitor ICU Database^®^: a cloud-based national registry for adult intensive care unit patients in Brazil

**DOI:** 10.5935/0103-507X.20170062

**Published:** 2017

**Authors:** Fernando Godinho Zampieri, Márcio Soares, Lunna Perdigão Borges, Jorge Ibrain Figueira Salluh, Otávio Tavares Ranzani

**Affiliations:** 1 Research Institute, HCor-Hospital do Coração - São Paulo (SP), Brazil.; 2 Instituto D’Or de Pesquisa e Ensino - Rio de Janeiro (RJ), Brazil.; 3 Epimed Solutions - Rio de Janeiro (RJ), Brazil.; 4 Intensive Care Unit, Department of Clinical Emergencies, Hospital das Clínicas, Faculdade de Medicina, Universidade de São Paulo - São Paulo (SP), Brazil.

**Keywords:** Hospital information systems, Database, Intensive care units, Sistemas de informação hospitalar, Base de dados, Unidades de terapia intensiva

## Abstract

**Objective:**

To describe the Epimed Monitor Database^®^, a Brazilian intensive care unit quality improvement database.

**Methods:**

We described the Epimed Monitor^®^ Database, including its structure and core data. We presented aggregated informative data from intensive care unit admissions from 2010 to 2016 using descriptive statistics. We also described the expansion and growth of the database along with the geographical distribution of participating units in Brazil.

**Results:**

The core data from the database includes demographic, administrative and physiological parameters, as well as specific report forms used to gather detailed data regarding the use of intensive care unit resources, infectious episodes, adverse events and checklists for adherence to best clinical practices. As of the end of 2016, 598 adult intensive care units in 318 hospitals totaling 8,160 intensive care unit beds were participating in the database. Most units were located at private hospitals in the southeastern region of the country. The number of yearly admissions rose during this period and included a predominance of medical admissions. The proportion of admissions due to cardiovascular disease declined, while admissions due to sepsis or infections became more common. Illness severity (Simplified Acute Physiology Score - SAPS 3 - 62 points), patient age (mean = 62 years) and hospital mortality (approximately 17%) remained reasonably stable during this time period.

**Conclusion:**

A large private database of critically ill patients is feasible and may provide relevant nationwide epidemiological data for quality improvement and benchmarking purposes among the participating intensive care units. This database is useful not only for administrative reasons but also for the improvement of daily care by facilitating the adoption of best practices and use for clinical research.

## Abstract (pt)

**Objetivo:**

Descrever a Epimed Monitor ICU Database^®^, uma base de dados brasileira cujo objetivo é a melhora da qualidade nas unidades de terapia intensiva do país.

**Métodos:**

Descrevemos a Epimed Monitor ICU Database^®^, inclusive sua estrutura e seus dados principais. Com utilização de estatística descritiva, apresentamos dados informativos agregados das admissões às unidades de terapia intensiva entre os anos de 2010 e 2016. Descrevemos também a expansão e o crescimento da base de dados juntamente da distribuição geográfica das unidades participantes no Brasil.

**Resultados:**

Os dados principais da base de dados incluíram informações demográficas, parâmetros administrativos e fisiológicos, assim como formulários específicos de relato para obter dados detalhados, com relação ao uso dos recursos da unidade de terapia intensiva, episódios infecciosos, eventos adversos e uma lista de verificação para adesão às melhores práticas clínicas. Até o final de 2016 tomou parte desta base de dados um total de 598 unidades de terapia intensiva para pacientes adultos, localizadas em 318 hospitais, perfazendo 8.160 leitos de terapia intensiva. Em sua maioria, as unidades participantes se localizavam em hospitais privados da Região Sudeste. O número anual de admissões apresentou um crescimento neste período, com predominância de admissões clínicas. A proporção de admissões em razão de doença cardiovascular diminuiu, enquanto as admissões por sepse ou infecções se tornaram mais comuns. Severidade da doença (*Simplified Acute Physiology Score* - SAPS 3 - 62 pontos), idade (média = 62 anos) e mortalidade hospitalar (cerca de 17%) permaneceram razoavelmente estáveis durante o período.

**Conclusão:**

Uma grande base de dados de pacientes críticos privados é viável e pode oferecer dados epidemiológicos abrangentes e relevantes para fins de melhoria da qualidade e comparação de resultados entre as unidades de terapia intensiva participantes. A base de dados é útil não apenas por razões administrativas, mas também por melhorar os cuidados diários, ao facilitar a adoção das melhores práticas e pode também ser utilizada em pesquisas clínicas.

## INTRODUCTION

The development of high-quality clinical databases is widely recognized as a necessity in the current field of critical care to evaluate outcomes and the process of care of critically ill patients. In a scenario of increasing complexity of care and rising costs in critical care delivery, such databases allow for performance evaluation of intensive care units (ICU) and are a rich source of data for clinical research^(1,2)^ as well as benchmarking.^(2)^ With this purpose, several intensive care registries, both non-commercial and commercial databases, have been developed in different countries.^(2-5)^ Most databases collect clinically relevant data on patient demographics, comorbidities, acute illnesses, diagnoses, severity-of-illness scores, treatments, adherence to best practices and outcome measures (e.g., mortality, length of stay (LOS), readmissions, ICU-related complications and infections). Typically, these databases aim to provide managerial and quality information for intensivists and hospital managers, allowing for assessment of risk-adjusted outcomes and clinical data to support the decision-making process at the ICU level and ultimately, allowing for benchmarking through blind comparison with aggregate or individualized data from other ICUs.

There are several examples of broad databases of critically ill patients. The Australian and New Zealand Intensive Care Society (ANZICS) now contains clinical and outcomes data from more than 1 million ICU patients, allowing users to access local ICU data and periodically access benchmarking data.^(1,3)^ The Intensive Care National Audit & Research Center (ICNARC)^(4)^ has consecutively enrolled ICU patients from the clear majority of ICUs in the UK since 1996. The NICE (Netherlands Intensive Care Evaluation) enrolls more than 80,000 consecutive adult ICU patients every year from almost all ICUs in the country.^(5)^

In addition to national, organizational-based systems, large private datasets are also available. A main example of these systems is the APACHE Outcomes system^(6)^ (created by the fusion of Project IMPACT^(7)^ with APACHE^(8)^), which is the most traditional database in the US. A third type of database includes both private and open-access databases of critically ill patients and is more focused on providing data for clinical research. Examples of this type of system include the High-Density Intensive Care (HiDenIC)^(9)^ database, which includes data from all critically ill patients admitted to one of the eight ICUs at University of Pittsburgh Medical Center (UPMC, Pittsburgh, PA) and the Medical Information Mart for Intensive Care III (MIMIC III) database, which includes data from over forty thousand patients admitted to ICUs in the Beth Israel Deaconess Medical Center betwen 2001 and 2012.^(10)^ All these ICU databases have been increasingly used in ICU epidemiology and outcomes research, as the bulk of publications in the field demonstrate.

In recent years, the development and growth of the Epimed Monitor Database^®^ (a cloud-based ICU performance management system) has accrued data on more than 1,300,000 ICU admissions in Brazil since 2009 and now covers approximately 30% of all adult ICU beds in the country. This represents an opportunity to generate relevant clinical studies to increase knowledge on the epidemiology of critical illness in Brazil^(11-13)^ and to evaluate specific risk factors for poor outcomes.^(14)^ In addition to being a tool for ICU management, such databases are in a unique position to allow for a better understanding of secular trends as well as trends in particular diseases (e.g., rare diagnoses, pandemics).

The aim of the present manuscript is to describe the Epimed Monitor ICU Database^®^ and its potential for use in clinical research.

### Database description

#### Definition of intensive care unit

The definition of ICU comes from the Brazilian National Definition, supported by both the *Associação Brasileira de Medicina Intensiva* (AMIB) and the *Agência Nacional de Vigilância Sanitária* (ANVISA), which can be summarized as follows: "unit dedicated exclusively to delivering care to critically ill patients who require the continuous care of health workers and the use of dedicated devices and technologies that are necessary to adequately diagnose, monitor and treat their conditions".^(15,16)^ Adult ICUs typically admit patients 18 years of age or older, but they may opt to admit patients between 15 to 17 years of age.

To comply with current regulations, each unit must have at least one coordinator for each section: a general unit coordinator, physician, nurse and physiotherapist coordinators. The ICU coordinator must be board certified in critical care. Every ICU in Brazil is required to have an attending physician present in the unit at all times, not including trainees. The recommendation is that at minimum, one attending physician, one nurse and one physiotherapist should be present for every 10 beds, and one nursing assistant should be present for every 2 beds. General auxiliary staff and structural conditions are similar to international guidelines.

#### Participation in the Epimed Monitor ICU Database^®^

Participation in the Epimed Database^®^ is voluntary and regulated by a commercial contract with an information technology company (Epimed Solutions^®^) that is responsible for the development, updates, security and backup of all processes. Most units included in the Epimed Monitor^®^ ICU are adult, pediatric or neonatal units. There are few high-dependency units. This report focuses on the adult critical care network.

#### Data entry and data ownership

All entered data originate from a structured and hierarchical electronic case report form (eCRF) that has a basic compulsory data frame, allowing customizations for some units or networks. Data are gathered by integration with the hospital's electronic (medical and/or administrative) records (EHR) and manual data entry. In most cases, each ICU has a dedicated case manager who is responsible for entering every consecutive patient into the database. This position receives dedicated training by the company, with periodic updates and feedback by mail. Online and live training also occurs along with regular (at least bimonthly) personal meetings with users. Cases are usually entered prospectively, except when patients are admitted on the weekends or if a patient dies or is discharged in less than 24 hours. On these occasions, if they are not entered prospectively in the database, charts are reviewed, avoiding selection bias or missing data. For specific eCRF sections such as hospital acquired infections, adverse events or daily checklists, other teams may be responsible for data entry.

Each entry is assigned a unique identifier. This unique identifier follows the order of the whole national database and is not grouped at the unit or hospital level. Readmissions within the same hospitalization or after hospital discharge always generate a new unique identifier number.

The database is structured to have active controls to guarantee data quality and data checking. To avoid processing errors, which encompasses coding and data entry steps, the definitions and labels of each variable are clearly stated in the eCRF and are also available in a PDF sheet that is easily accessible on the online platform. To address possible errors during data entry, the system provides checks during the data entry process ("interactive checking"). Conditional filling is also present for some specific variables. Unit coordinators and case managers can check the pattern of incomplete cases, such as the percentage of incompleteness, during a selected period of admission. Offline checks can occur at random depending on the demand for each unit and for database updates and improvement.

Each participating ICU has direct access only to its own data entered in the database. In the context of clinical research, data from units interested in participating in research are gathered after appropriate approval from each center's ethics committee, following the Brazilian guidelines for research. The steering committee of the research team eventually analyzes all data and creates a manuscript for publication.

#### eCRF structure

The eCRF is hierarchically structured and includes unique datasheets for time-independent variables and multiple datasheets for time-dependent variables. Unique datasheets refer to demographic data, comorbidities, admission diagnosis, acute physiologic data (in the first hour and at 24 hours after admission), need for organ support (at admission, in the first hour, and after 24 hours) and the presence of complications at ICU admission. Each data entry in the database is followed by a calendar date. Table 1 shows the core data for each admission.

**Table 1:** Core data for adult patients

Demographic data	Admission data	Device use and physiological data
Age	Main diagnosis and admission type	Use of vasopressors
Sex	Source	Use of mechanical ventilation
Comorbidities	Presence of infection	Laboratory data

Demographic data comprises unique patient identifiers, age, whether this instance is a readmission during the same hospitalization (and whether this readmission occurred within 24 hours of ICU discharge), weight, height and bed number. Comorbidities comprise all comorbidities from the Charlson Comorbidity Index^(17)^ and additional comorbidities that may be useful for risk assessment and stratification for specific conditions (e.g., stroke, coronary disease). A measurement of performance status in the week prior to ICU admission adapted from the Eastern Cooperative Oncology Group (ECOG)^(18)^ is also collected.

Admission is classified as medical, elective surgery or emergency/urgent surgery. The source of admission is also recorded. Based on the initial classification, a list of main reasons for admission is available, comprising several categories (Table 2). Within each main category, there is a list of pre-specified diagnoses. A codification based on the ICD-10 is also available. Dynamic datasheets are generated if a new diagnosis is made (secondary diagnosis).

**Table 2:** Diagnostic categories

Medical admissions	Surgical admissions
Cardiovascular	Orthopedic surgery
Infection/sepsis	Cardiac surgery
Neurologic	Combined cardiac surgery
Respiratory	Congenital cardiac surgery
Gastrointestinal	Vascular surgery
Renal	Neurosurgery
Hematologic	Liver/biliary tract/pancreas surgery
Oncologic	Gastric surgery
Endocrine/metabolic	Esophagus surgery
Allergic and rheumatologic diseases	Bariatric surgery
Shock (except sepsis)	Colon surgery
Multiple organ failure	Other abdominal/retroperitoneal surgeries
Monitoring	Lung/trachea surgery
After cardiopulmonary resuscitation	Other thoracic surgery
Palliative care	Head and neck surgery
Non-surgical trauma	Prostate surgery
Brain death	Urinary tract surgery
	Gynecologic/breast surgery
	Solid organ transplantation
	Endocrine gland surgery
	Other elective surgeries
	Other urgent surgeries
	Surgical Trauma
	Skin and soft tissues surgery
	Hernia or abdominal wall repair
	Ophthalmologic surgery
	Male genital organs surgery
	Surgical procedures
	Invasive procedures
	Cardiac invasive procedures
	Endovascular procedures

The need for organ support at admission, during the first hour and within the first 24 hours of ICU admission is also recorded. These data include the use of vasopressors and inotropes, mechanical ventilation (invasive and non-invasive) and renal replacement therapy. The presence of complications such as cardiac arrest and acute renal failure is also recorded. Laboratory and physiological data are also recorded both for the first hour and at 24 hours after admission (Table 3).

**Table 3:** Laboratory and physiological data available

Vital signs	Blood analyses	Blood gas
Systolic blood pressure	Leukocytes	pH
Diastolic blood pressure	Platelet	PaO_2_
Respiratory rate	Creatinine	PaCO_2_
Heart rate	Urea	
	Bilirubin	
	Lactate	

pH - acidity; PaO_2_ - partial pressure of oxygen; PaCO_2_ - partial pressure of carbon dioxide.

Daily data are updated regarding new organ support, invasive procedures, specific organ support such as extracorporeal membrane oxygenation or use of an intra-aortic balloon pump, and nurse workload, as assessed by the Nursing Activities Score (NAS). There are checklists for sedation, invasive device care, mechanical ventilation, ulcer pressure prevention, sepsis bundles and bundles for prevention of hospital-acquired infections. Care goals, including the decision to initiate exclusive palliative care, may also be recorded in the web version or mobile application. Applications for the management of checklists, Sequential Organ Failure Assessment (SOFA) scores and daily goals are also provided in Android^®^ and iOS^®^ versions. These applications may be used for data entry by the healthcare team during bedside activities.

#### Quality indicators

Core quality indicators are those recommended by ANVISA^(16)^ and the European Society of Intensive Care Medicine (ESICM) task force^(19)^ to evaluate ICU performance. The following indicators are collected in the database: ICU and hospital mortality rate, standardized mortality ratio (SMR) according to the score selected, early unplanned ICU readmissions (<24 hours and 48 hours after discharge), ICU and hospital LOS, bundle of prevention measures related to hospital-associated infections, incidence rate of specific nosocomial infections (e.g., ventilator-associated pneumonia, central line-associated bloodstream infection, catheter-associated urinary tract infection), qualitative and quantitative evaluation of nurse workload, and monitoring of adverse events. The Epimed Monitor^®^ Database provides surveillance for incidents and adverse events such as transfusion-related incidents and complications, drug-induced adverse events, unintended extubation, catheter dislodgment and pressure ulcers.

#### Scoring systems

Severity scores are calculated for every patient from compulsory data. The SAPS 3^(20)^ is mandatorily collected following ANVISA and AMIB recommendations. The calibration of scores is periodically checked for the necessary updates. The general SAPS 3 equation provides a better calibration for the database and is thus used in the system for benchmarking purposes.^(21)^ Some additional scores are also available in the system, including the aforementioned Charlson Comorbidity Index,^(17)^ the SAPS II, the Acute Physiology and Chronic Health Evaluation (APACHE) II^(22)^ and IV^(8)^ and the SOFA.^(23)^ The SOFA score can be calculated daily using the dynamic datasheets or the mobile applications.

#### Length of stay prediction

One important piece of additional information for assessing each unit's effectiveness is the unit's LOS, which is still the best marker of resources available, and it is frequently employed to obtain efficiency matrices for ICUs. The Epimed Monitor system collects data on LOS and provides clinical guidance for future admissions. For each diagnostic category and from demographic information, an LOS estimate is calculated by the system; however, instead of simply reporting the predicted number (which could in turn create a bias by "pressuring" the physician to discharge the patient from the unit), the system indicates whether landmark periods have passed (for example, if the patient exceeded the 75^th^ percentile of LOS for that specific diagnosis) and provides an individualized risk of prolonged length of stay (LOS longer than the 90^th^ percentile of LOS for each given diagnosis).

## RESULTS

**Figure 1:**
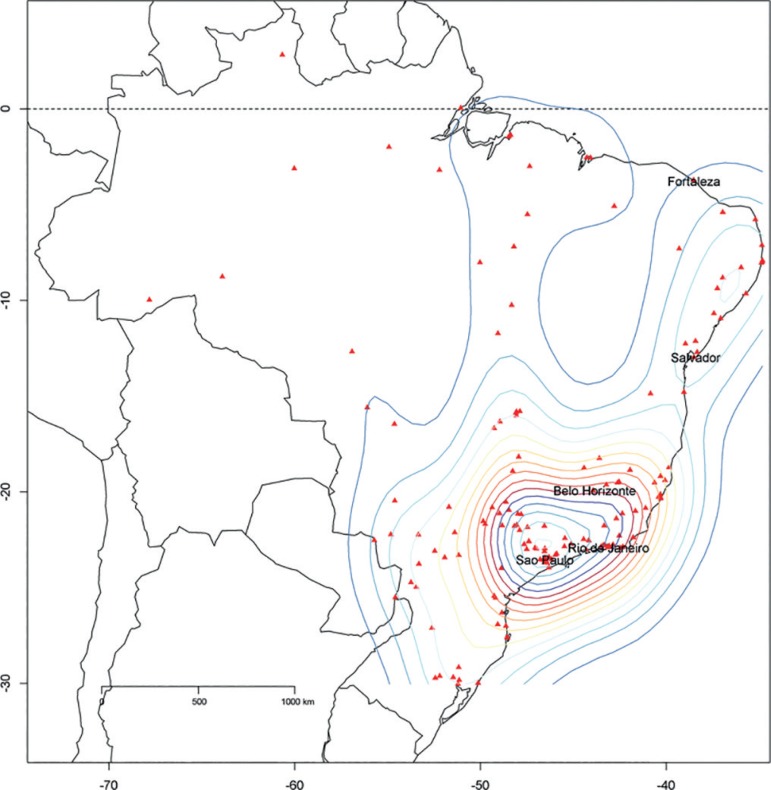
Cities with units using the Epimed Monitor in Brazil. A 2D stat density plot is overlaid on the map. The density plot provides a visual representation of the distribution of data over a continuous interval. (Therefore, it is a variation of a histogram using kernel smoothing.) In this figure, the density plot is presented in two dimensions according to the latitude and longitude of the participating intensive care units.

**Figure 2:**
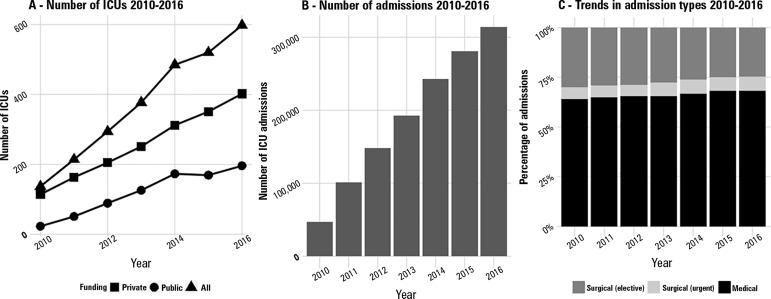
Trends in numbers of intensive care units, numbers of admissions and admission types 2010 - 2016. ICU - intensive care unit.

The geographical distribution of participating ICUs at the end of 2016 (598 units in 318 hospitals, totaling 8,160 ICU beds) is shown in figure 1. All five regions of Brazil are represented in the database, with units concentrated in the southeastern region. The number of ICUs has been increasing each year, with a predominance of private ICUs over public units (Figure 2A). As a consequence of the increase in participating ICUs, the number of admissions per year is also rising, with over 300,000 admissions registered in 2016 (Figure 2B). Male gender is slightly predominant (50.6%). The mean patient age was 62 years (standard deviation 20 years) during this period, with very small fluctuations.

Most admissions are for medical surgeries, followed by elective surgeries. Urgent surgeries account for less than 7% of all admissions in all years examined. From 2010 to 2016, the proportion of medical admissions slightly increased (Figure 2C).

**Figure 3:**
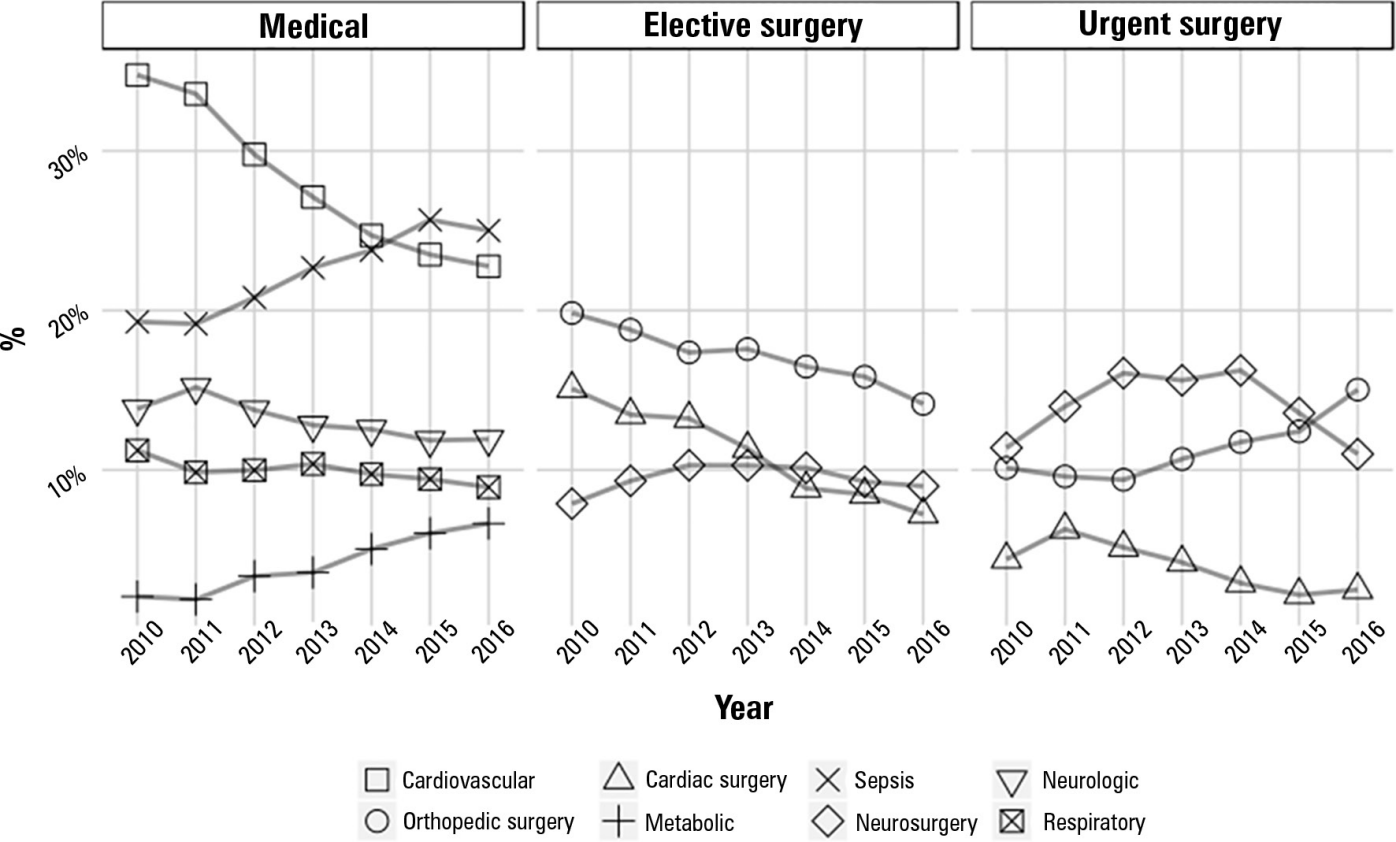
Admissions 2010 - 2016 by type.

Trends in the main reasons for admission by each admission type are shown in figure 3. For clarity purposes, only the diagnostic reasons that corresponded to more than 3% of all admissions are shown. A decline in admissions due to cardiovascular reasons is evident, which is followed by an increase in the number of admissions due to infection/sepsis. The percentage of admissions due to metabolic reasons is also increasing. The proportional number of elective orthopedic surgeries has decreased, while the number of urgent orthopedic surgeries has increased. The proportion of admissions due to cardiac surgery has also decreased.

**Figure 4:**
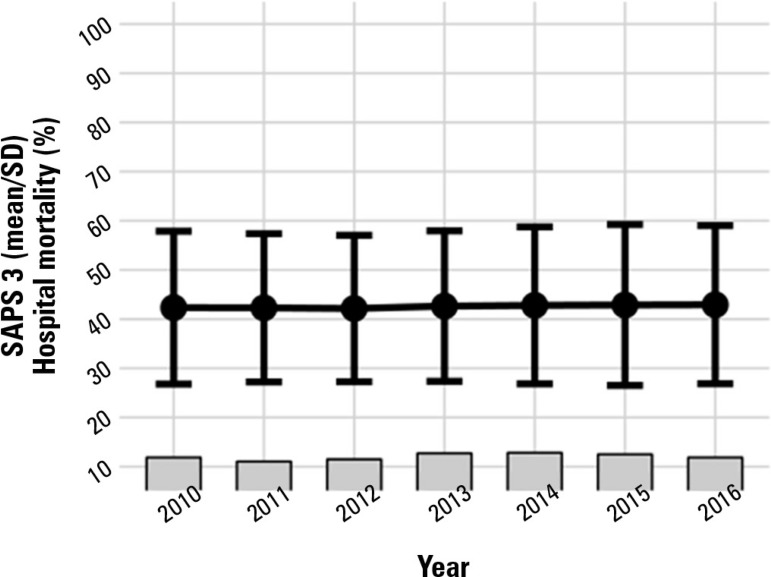
Simplified Acute Physiology Score 3 score and hospital mortality from 2010 to 2016. SAPS 3 - Simplified Acute Physiology Score 3.

**Figure 5:**
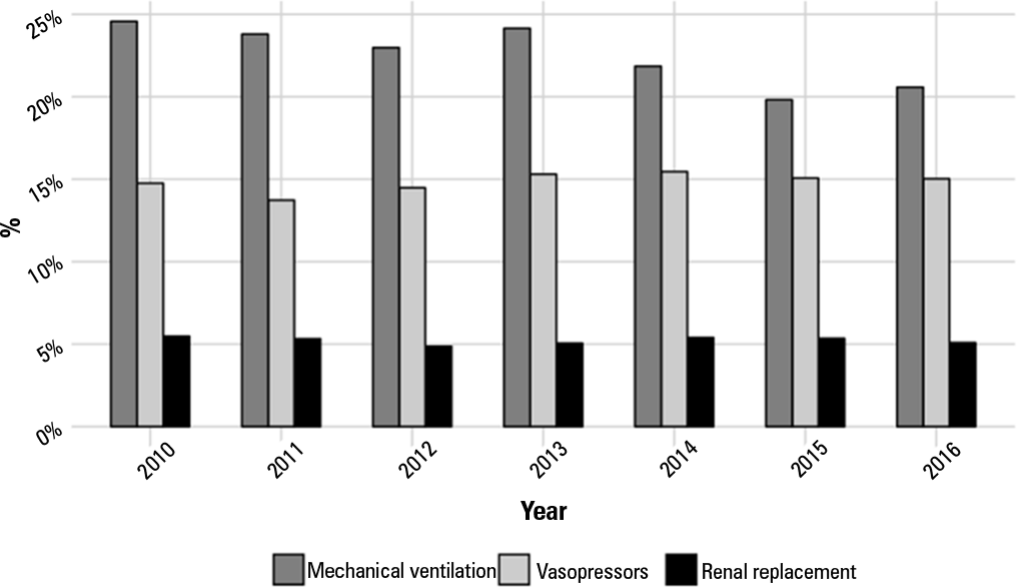
Use of organ support 2010 - 2016.

Illness severity was mostly constant from 2010 to 2016 (mean SAPS 3 ~ 42 points; standard deviation ~16; Figure 4). Hospital mortality remained at approximately 17 - 18% (Figure 4). The mean standardized mortality ratio peaked in 2013 (1.27) and reached its lowest level in 2016 (1.09; both using the SAPS 3 global equation). ICU mortality was approximately 11-12%. The use of vasopressors and renal replacement therapy remained constant in the last seven years (15% and 5%, respectively), while a small decrease in the use of invasive mechanical ventilation occurred, especially in the last two years (from approximately 25% to 20%; Figure 5).

## DISCUSSION

In the present manuscript, we describe the structure, the core data available and the additional report forms of a private electronic database of critically ill patients in Brazil. While the database was initially designed for ICU quality and performance, it has grown to encompass other important tools for both ICU managers and healthcare teams. Therefore, in addition to allowing benchmarking and providing data on trends on bed occupancy and resource use, the system may be useful at the bedside, allowing the adoption of best practices and bundles. Additionally, it has proven to be a reliable tool for observational prospective research.

Inspection of the results highlights several important trends in critical illness in Brazil, such as a decrease in cardiovascular admissions and an increase in sepsis admissions. Additionally, despite changes in participating units and the national comprehensiveness of the registry, illness severity has remained largely unchanged, but a decrease in the use of mechanical ventilation was observed, potentially indicating a change in patient profiles and resource use in Brazilian ICUs. While the mix of participating units may be at least partly responsible for these fluctuations, one of the key aspects of the Epimed Monitor Database in the future will be legacy data and the capability to perform trend analyses in the near future. Are Brazilian critically ill patients becoming sicker or more fragile? Is the number of critically ill oncology patients increasing? Has the frequency of severe dengue or influenza cases changed? Are there regional variations in care that should be considered by healthcare authorities? All these answers may come from a large broad patient registry. The current ongoing "*UTIs Brasileiras*" project (www.utisbrasileiras.com.br) is the first major effort to obtain reliable epidemiological data on Brazilian UTIs using the database, making it available for healthcare professionals, patients, families, policy makers and society in general. Additionally, the database is currently expanding to Latin America and Europe while keeping the same core data concepts, which may allow for future collaborations with other networks such as ICNARC^(24)^ and international benchmarking.

The database has already proven its usefulness in relevant observational studies. The ORCHESTRA study^(13)^ was a large observational cohort including 2013 data from 78 ICUs participating in the Epimed Monitor System. The authors used data on organizational features at the unit level and assessed their association with outcomes and found that the number of protocols was associated with mortality (*odds ratio* - OR 0.944; 95% confidence interval - 95%CI 0.904 - 0.987 for each existing protocol). Additionally, higher protocol use was associated with more efficient resource use. It should be highlighted that the study also provided data on the habits of the participating ICUs. For example, only 46% of all participating units used daily checklists, and fewer than 25% had a board certified intensivist present at all times.

A sequential subanalysis that also included data from the Epimed Monitor focused on critically ill patients with cancer.^(25)^ This analysis confirmed the important role of organizational factors, finding that the presence of clinical pharmacists in the ICU and the number of protocols and daily meetings between oncologists and intensivists for care planning were associated with lower mortality. Additionally, in a subsequent analysis of the main ORCHESTRA study, the authors evaluated the association between family visitation policies and unit standardized mortality ratio and found an association between family visits and better unit performance.^(26)^ All these reports suggest that a solid high-quality prospective database of critically ill patients is essential to assess the impact of organizational and behavioral policies in the critically ill.

The database was also utilized to assess and validate specific risk factors for mortality in critically ill patients. For example, although preliminary data suggested that performance status could be associated with worse outcomes in critically ill patients,^(27)^ there was no high-quality multicenter evidence to support this. The unique features of the Epimed Monitor, including the measurement of ECOG performance status and other proxies of functionality (such as age and comorbidities), allowed for a larger study that confirmed the important association between worse performance status and higher mortality after ICU admission.^(14)^

Some limitations of the system should also be mentioned. Despite minimal fixed core data, there is some variability in the diversity of the data collected at each ICU. Additionally, data resolution is limited, with most information concentrated in the first 24 hours after ICU admission. This is different from other databases that may display high-resolution (sometimes hourly) data for selected patients. Finally, the system depends on private funding for maintenance and therefore is not freely available. Consequently, there is a predominance of private units, which limits the system's capability to represent the full picture of critical care in Brazil.

## CONCLUSION

The Epimed Monitor ICU Database^®^ is a fast-growing database of clinical and administrative data from over 1,300,000 critically ill Brazilian patients. Despite limitations in availability, the large number of included intensive care units allows one to assess the picture of critical illness in Brazil, thereby fostering clinical research.
